# Mechanical alterations of bitumens with different crude oil origins through polymer modification

**DOI:** 10.1038/s41598-026-53636-8

**Published:** 2026-06-18

**Authors:** Anıl Baykara, Mehmet Yılmaz, Erkut Yalçın, Ahmet Münir Özdemir, Taner Alataş

**Affiliations:** 1 Civil Engineering Department, Firat University, Elazig, Turkey; 2 Civil Engineering Department, Bursa Technical University, Bursa, Turkey; 3 Beytek Software Electronics Construction and Trade Co. Ltd, Firat Technopark, Elazig, Turkey; 4 Ingenible AI Software Informatics and Engineering Trade Co. Ltd, Firat Technopark, Elazığ, Turkey

**Keywords:** Hot mix asphalt, SBS, SEBS, Bitumen origin, Mechanical performance, Chemistry, Engineering, Materials science

## Abstract

Hot mix asphalt (HMA) performance is significantly influenced by the chemical variability of bitumen, which is closely linked to crude oil origin. These source-dependent variations also affect the response of bitumen to polymer modification. This study investigates the mechanical performance of asphalt mixtures produced using two bitumens with the same penetration grade but obtained from different refineries (Turkey–Batman and Iraq–Lanaz), modified with two styrene-based polymers: styrene-butadiene-styrene (SBS) and styrene-ethylene-butadiene-styrene (SEBS). Polymer-modified binders (2%, 3%, and 4%) were prepared and evaluated through Marshall stability, indirect tensile strength (ITS), stiffness modulus (ITSM), fatigue, and dynamic creep tests. The results demonstrated that bitumen origin plays a decisive role in both binder behavior and mixture performance. SEBS modification produced superior stability and deformation resistance in mixtures with Iraqi bitumen, whereas SBS modification provided the highest stability and stiffness improvement in mixtures with Batman bitumen. Fatigue and creep analyses indicated that SEBS-modified mixtures, particularly with 3% SEBS, achieved the longest fatigue life and highest resistance to permanent deformation. Overall, the study highlights that the interaction between binder origin and polymer type critically governs the performance of polymer-modified asphalts, and that optimal polymer dosage varies depending on the source characteristics of the bitumen.

## Introduction

The performance of HMA is seriously affected by factors such as increasing axle loads, high and low temperatures, freezing and thawing, and precipitation, resulting in permanent deformation, crack formation, and delamination^1,2^. Bituminous binders, which are present in relatively low proportions (4–7%) within HMA, are highly responsible for the final performance of the pavement^3–6^. As is well known, bitumen is produced through the refining of crude oil and presents itself as a complex material with a highly intricate chemical structure. Changes in the source of bitumen result in variations in its physical and chemical properties. These variations also affect the performance of the binder and, ultimately, the mixture. Even if they have the same penetration grade, differences in source can lead to variable results.

The chemical composition of binder depends on the source of petroleum, and the production process (flat working, blowing, etc.) also has a significant effect^7^. The chemically intricate structure of binder presents a challenge in elucidating the behavioral system during its characterization. For years, researchers have been striving to clarify the chemistry of binder, but despite their best efforts, they have yet to achieve a clear result^8,9^.

Pei and his team analyzed seven different binders in their study and found that the source of the binder had a significant effect on ageing behaviour, while the effect of the penetration grade was relatively limited^10^. Michalica et al. investigated the chemical and rheological properties of two different binders sourced from Russia and Canada. Chemical, physical, and rheological tests were performed on unaged, RTFOT-aged, and PAV-aged samples. The results showed that the binder sourced from the Canadian region had higher asphaltene, higher heteroatoms, and lower aging sensitivity. This was also reflected in the rheological tests, where it was found that the physical properties of Canadian bitumen degraded more slowly in terms of aging^9^. In another study, Wu et al.^11^ investigated how the chemical composition and microstructure of bitumen affect workability. Bituminous binders sourced from three different refineries were subjected to chemical analysis, revealing source-based differences. The results showed that bituminous binders with higher sulfur content experienced greater increases in viscosity after aging, and that there were significant correlations between bitumen chemistry and mechanical properties. It has also been reported that the source of bitumen is important for short- and long-term performance, particularly moisture damage resistance. A study claimed that the quality control standards used did not adequately account for differences in bitumen sources^12^. The responses of binders obtained from different sources to modification may also vary. In a study, antioxidants were added to bitumen obtained from different sources, and the chemical, physical, and rheological properties of the binder were investigated. The results obtained indicate that the selection of appropriate raw materials and the use of additives are important factors in improving asphalt performance and extending pavement life. Zhai et al. investigated the influence of physicochemical and thermodynamic parameters on cohesion properties utilizing nine distinct bitumen sources from different refineries. Their findings demonstrated that the bitumen source significantly impacted the performance of asphalt pavements^13^. Another study reported that the method of obtaining bitumen at the refinery also has an impact on bitumen properties^14^.

Bitumen material demonstrates viscoelastic material properties, exhibiting a combination of viscous liquid behavior at elevated temperatures or extended loading times and elastic solid behavior at low temperatures or brief loading times^15^. This behavior of binder contributes to the formation of rutting deformation in flexible pavements at high service temperatures and under the influence of slow-moving and heavy vehicles^16^. To mitigate the deleterious effects of high-temperature deformation, various additives have been employed^17^. The modification process aimed at enhancing the performance of pavements involves the introduction of additives to binder. This modified binder is subsequently utilized in the plant’s production process.

Polymers have been extensively utilized as modifiers due to their capacity to transfer their properties to asphalt^18^. For instance, the enhancement of rutting resistance in polymer-modified binders diminishes their fatigue and cracking resistance, as well as their thermal sensitivity^19,20^. SBS is a block copolymer and the most commonly used additive in asphalt modification. Structurally, it contains hard styrene domains embedded in a soft butadiene matrix and belongs to the thermoplastic elastomer class. SBS modification is roughly the mechanical breakdown of the polymer within the asphalt binder matrix under high shear conditions. After cooling from the modification temperature to room temperature, the styrene segments of SBS aggregate, and the original network structure is reestablished. In this state, the butadiene phase swells along with the bitumen molecules^21–25^.

The compatibility of SBS with asphalt at the molecular level is a vital aspect that influences its efficacy as an asphalt modifier. When SBS is completely miscible with asphalt, the characteristics of the asphalt are anticipated to remain predominantly unchanged with the incorporation of moderate quantities of the polymer. In contrast, if SBS and asphalt exhibit significant incompatibility, the polymer’s dispersed particles may merge into a macroscopically distinct phase, which could jeopardize the structural integrity of the modified asphalt. The chemical composition and polarity of SBS render it an appropriate modifier for various asphalt types, thereby diminishing the chances of severe solubility and phase separation issues. Nevertheless, similar to all unsaturated rubbers, SBS is prone to aging, which restricts the potential for recycling the pavement once its lifecycle concludes. Its limited resistance to heat, oxidation, and ultraviolet radiation can render SBS less advantageous^26,27^.

In recent years, saturated polymer additives that could be alternatives to SBS have become popular among researchers. Styrene-(ethylene-co-butylene)-styrene triblock copolymers (SEBS) produced by direct hydrogenation of SBS are considered a reasonable alternative option^28,29^. The saturation of double bonds is a notable advantage of SEBS compared to SBS. Additionally, SEBS has lower polarity than SBS^30^. Asphaltenes are known to be the most polar components in the binder, and P(EB) blocks, which are less polar than PB blocks, are associated with this issue.

Under these conditions, it can be asserted with certainty that SEBS is capable of blending with maltenes, but not entirely with asphaltenes^31^. Nevertheless, it is essential to acknowledge that these observations may not be universally applicable to all asphalt formulations.

Polacco et al. prepared SEBS-modified bitumens and found that stable binders in terms of storage stability can be obtained by using 4 wt% SEBS. According to the authors, a “filler effect” occurs when SEBS is used at low rates, and a higher performance effect occurs when the rate is increased. However, in this case, there are also negative effects in terms of storage stability^31^. In a separate study, Elseifi et al. examined the rheological properties of bituminous binders with varying proportions of SBS and SEBS, finding that SEBS exhibited higher rutting resistance compared to SBS. However, SBS demonstrated superior fatigue resistance at intermediate temperatures^32^. Becker et al. discovered that PMAs based on SEBS, which were created using low penetration asphalt, exhibited greater stability compared to those made with SBS. They asserted that PMAs formed by blending asphalt with SEBS through a straightforward high shear process are stable^27^. The number of studies on SEBS modified asphalt is relatively small and more research is needed due to the aging resistance of SEBS, environmental considerations, advantages over SBS, etc.

In this study, bitumens with identical penetration grades (160/220) but originating from two refineries with markedly different chemical characteristics LANAZ (Iraq) and TÜPRAŞ Batman (Turkey) were modified using styrene-butadiene-styrene and styrene-ethylene-butadiene-styrene polymers at 2%, 3%, and 4% dosages. By producing hot mix asphalt specimens with these polymer-modified binders, the research systematically isolates and compares the mechanical responses arising solely from source dependent variations in bitumen chemistry. This approach provides a unique framework that goes beyond conventional modifier-focused studies, enabling a clear identification of how the same polymer interacts differently with bitumens of distinct origins. Through this comparative methodology, the study offers a comprehensive and novel perspective on the role of bitumen source in determining the effectiveness of SBS and SEBS modifications, thereby filling a critical gap in understanding cross-country bitumen variability and its implications for asphalt mixture performance.

In other words, the novelty of this study lies not in the use of polymer modification itself, but in the comparative evaluation of binders with identical penetration grades obtained from different crude sources and their interaction with different polymer types (SBS and SEBS). While previous studies have largely focused on binder-level rheological properties, this study provides a mixture-level performance perspective, demonstrating how binder source and polymer type jointly influence key performance indicators such as stiffness, fatigue resistance, moisture susceptibility, and rutting resistance.

## Materials and methods

### Materials

The bitumens used in this study were obtained from two different refineries, namely Batman TÜPRAŞ (Türkiye) and Lanaz (Northern Iraq), representing binders derived from geologically and chemically distinct crude oil sources. The Batman refinery processes crude oils originating from southeastern Türkiye, which are typically associated with more complex and heavier hydrocarbon fractions [33]. In contrast, the Lanaz refinery processes crude oils from northern Iraq, which are known to exhibit different compositional characteristics and are refined under different processing configurations.

Such differences in crude oil origin and refining routes are known to influence the balance between saturates, aromatics, resins, and asphaltenes, as well as the overall colloidal structure of bitumen. These variations can significantly affect polymer compatibility, swelling behavior, and the formation of polymer networks within the binder. Therefore, the use of these two binders provides a robust framework for assessing how crude oil origin influences the performance of polymer-modified asphalt systems.

**Fig. 1:**
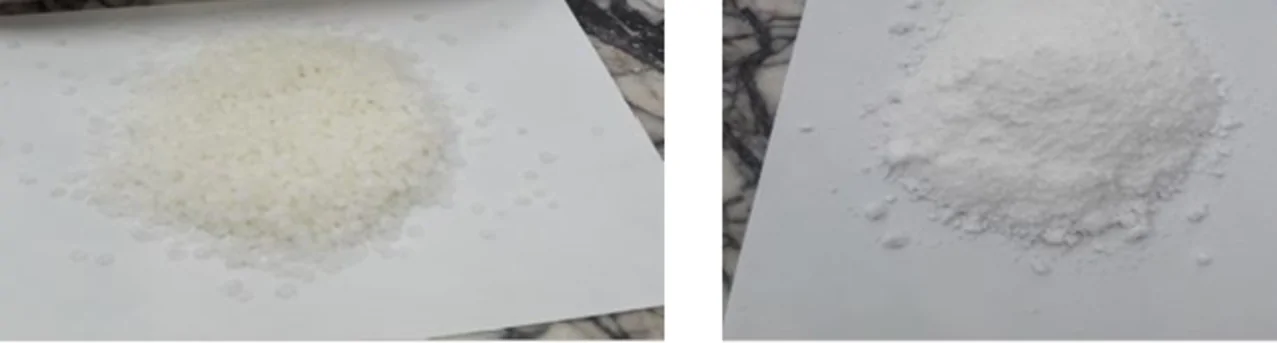
**a**) SBS and **b**) SEBS additives.

Pure bitumen was obtained from two different regions: from Batman Petroleum Refinery in Turkey, with 189 dmm penetration, a 42.2 °C softening point, and a viscosity of 262.5 cP at 135 °C and from Iraq, with 177 dmm penetration, a 40.65 °C softening point, and a viscosity of 237.5 cP at 135 °C. SBS and SEBS were obtained from Shell Chemical Company. The molecular structure, styrene/butadiene ratio, density, and stiffness of SBS are linear, 31/69, 0.94, and 70, respectively. The density, styrene content, tensile strength, and stiffness of SEBS are 0.88 and 1.25 g/cm³, 30%, 34 MPa and 75, respectively. Images of the additives are given in Fig. 1.

### Preparation of bitumen and mixtures with elastomer additives

In this research, modified binders were generated by incorporating two distinct elastomers at three varying proportions (2%, 3%, and 4%) into pure bitumen sourced from two different origins. The modified bitumen was obtained by mixing pure bitumen and additives at 180 °C for 60 min with a mechanical mixer with a speed of 1000 rpm. The modified bitumen preparation process was determined by reviewing previous studies^34^. The binder test results of pure and modified Batman and Iraqi bitumen are presented in Table 1. The results in Table 1 have been compiled from previous studies conducted by our research team^35,36^. In the study, the pure bitumen obtained from Batman was designated “B”, while the pure bitumen obtained from Iraq was designated “I”.

The data presented in Table 1 clearly demonstrate how the physical and rheological properties of binders obtained from two different sources Batman (Turkey) and Lanaz (Iraq) evolve when modified with SBS and SEBS polymers at varying dosages. Penetration values decrease consistently with increasing polymer content, indicating a systematic stiffening effect that is considerably more pronounced in SEBS-modified binders due to the polymer’s saturated structure and stronger interaction with the maltene phase. Correspondingly, the softening point rises with each increment of polymer dosage, with SEBS again producing the greatest increase, reflecting its superior ability to enhance high-temperature performance. Brookfield viscosity values at both 135 °C and 165 °C increase sharply, especially in SEBS blends, which not only corroborates the higher degree of structural modification but also explains the substantially elevated mixing and compaction temperature requirements observed for SEBS-containing binders. The Performance Grade (PG) classification further supports these trends: whereas SBS raises the PG of both base binders from their original levels (PG 52 for Batman and PG 58 for Iraq) to PG 64 at higher dosages, SEBS consistently advances the PG to PG 70, indicating a more robust improvement in rutting resistance. Differences between the two base bitumens also become apparent; the Iraqi bitumen, initially softer and more penetrable, exhibits a more dramatic response to polymer modification, while the Batman bitumen, being naturally stiffer, transitions more gradually across performance levels. Overall, the table highlights three critical findings: (i) bitumen origin substantially influences the effectiveness of polymer modification, (ii) SEBS induces greater improvements in high temperature stiffness and PG grade but also causes higher viscosity and processing temperatures, and (iii) the relationship between polymer dosage and binder enhancement is nonlinear, with the magnitude of improvement depending jointly on polymer type and base bitumen chemistry^9,21,31^.

**Table 1:** Properties of pure and modified binders^35,36^.

Binder	Penetration, dmm	Softening Point, °C	Brookfield Viscosity, cP, 135 °C/165°C	DSR-Complex Modulus, PG	Mixing Temperature Range, °C	Compaction Temperature Range, °C
I-Pure	188.67	42.2	262.5/100	PG 58	145.8–153.2.8.2	130.5–137.2.5.2
I-SBS2	136.90	43.1	325/112.5	PG 58	151.7–158.5.7.5	137.5–143.9.5.9
I-SBS3	128.47	47.55	425/150	PG 64	156.9–163.6.9.6	142.9–149.1.9.1
I-SBS4	126.20	52.4	500/162.5	PG 64	162.9–169.3.9.3	149.6–155.4.6.4
I-SEBS2	93.85	45.85	475/187.5	PG 64	164.5–172.1.5.1	148.7–155.7.7.7
I-SEBS3	78.20	48.45	650/275	PG 64	175.9–184.1.9.1	159.0–166.4.0.4
I-SEBS4	76.68	50.1	875/375	PG 70	190.7–199.1.7.1	173.2–180.9.2.9
B-Pure	177	40.65	237.5/87.5	PG 52	143.0–150.1.0.1	128.1–134.6.1.6
B-SBS2	119.63	42.45	362.5/125	PG 58	155.3–162.1.3.1	141.5–147.5.5.5
B-SBS3	107.53	46.7	475/187.5	PG 64	164.5–172.1.5.1	148.7–155.7.7.7
B-SBS4	98.57	52.5	550/200	PG 64	165.2–172.1.2.1	150.8–157.1.8.1
B-SEBS2	101.50	45.15	500/225	PG 64	169.0–177.7.0.7	150.8–158.8.8.8
B-SEBS3	84.87	48.1	687.5/300	PG 70	179.2–187.6.2.6	161.7–169.4.7.4
B-SEBS4	65.78	51.2	912.5/387.5	PG 70	187.0–195.1.0.1	170.1–177.5.1.5

In this study, I-Pure, B-Pure, I-SBS4, I-SBS4, I-SEBS3, B-SBS4, and B-SEBS3 binders were selected for the evaluation of mixture performance. The data obtained from previous conventional binder tests have shown that these mix types will exhibit superior performance.

**Fig. 2:**
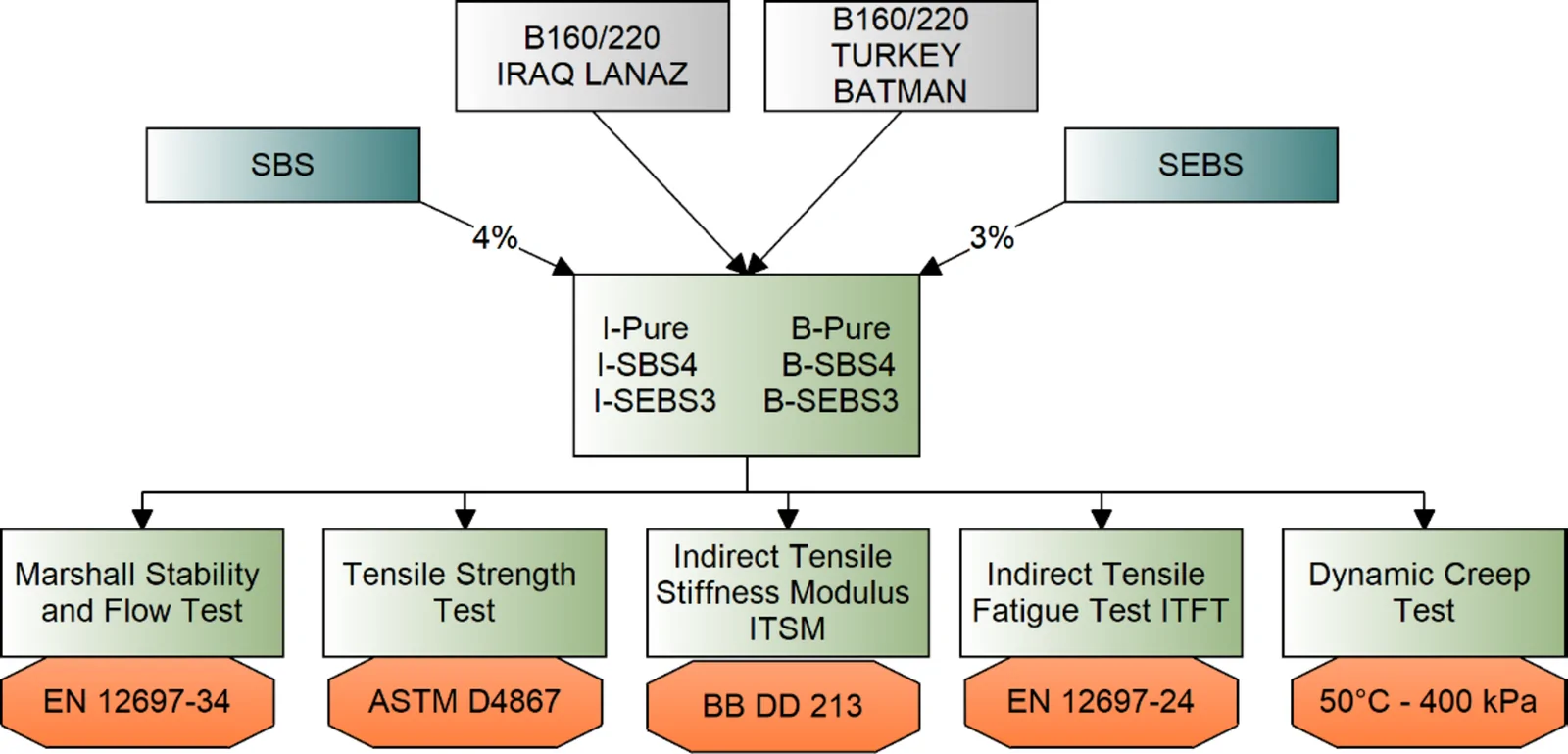
Experimental flowchart of the study.

The mixture specimens were prepared according to the aggregate gradation specified in Table 2, following the Superpave mix design methodology. The optimum binder content of the control mixture was determined as 4.67% based on volumetric design criteria. In order to eliminate the influence of binder content variability and ensure a consistent comparison, the same binder content was used for all modified mixtures. Polymer modification levels were defined as a percentage of the binder weight, and the modified binders were incorporated into the mixtures under controlled conditions. The mixtures were prepared at the appropriate mixing and compaction temperatures corresponding to the viscosity requirements of the binder. Compaction was carried out using a Superpave gyratory compactor to produce cylindrical specimens with uniform density and air void distribution. All specimens were compacted to the target number of gyrations in accordance with Superpave design procedures to achieve the desired air void content. After compaction, specimens were allowed to cool and stabilize at room temperature before testing. Prior to mechanical testing, conditioning procedures were applied in accordance with the relevant standards to ensure consistent moisture and temperature conditions across all samples. The experimental flow chart, which outlines the methodological framework of the study, is illustrated in Fig. 2.

All experimental tests were conducted using three replicate specimens for each mixture. The reported results represent the average values, and the variability of the data is expressed in terms of standard deviation. Error bars representing standard deviation have been included in the figures to provide a clearer assessment of data dispersion and repeatability.

**Table 2:** Aggregate gradation used in asphalt mixture.

Sieve size (mm)	19	12.5	9.5	4.75	2.36	1.18	0.6	0.3	0.15	0.075
**Pass (%)**	100	95	88	65	35	23	14	10	8	6

### Marshall stability and flow test (EN 12697-34)

Stability is characterized as the highest resistance to deformation, whereas yield refers to the deformation that takes place in the sample upon reaching the maximum load. In accordance with the standard, the heights of the samples that have been compressed and cooled are initially measured and documented. Subsequently, these samples are maintained in a water bath at a temperature of 60 ± 1 °C for a duration of 40 to 60 min. Additionally, the crushing jaw must be submerged in water at a temperature of 60 ± 1 °C for 30 min or placed in an oven at the same temperature of 60 ± 1 °C for 1 h. At the end of this period, the sample is removed from the water, placed in the center of the crushing jaw, and crushed at a constant speed of 50 mm/min. The Marshall quotient (MQ) is obtained by dividing stability by flow. A higher value indicates that the asphalt is more resistant to permanent deformation^37,38^.

Retained Marshall Stability (RMS) is a measure that expresses the effect of moisture on asphalt mixtures exposed to moisture conditions and corresponds to the durability of the asphalt. It is obtained by dividing the Marshall stability values of mixture samples conditioned by being kept in a water bath at 60 °C for 24 h by the normal Marshall stability values (unconditioned) of the samples.

### Resistance to moisture damage (tensile strength ratio) test

In this test, the resistance to moisture damage in HMA is measured. The test was conducted in accordance with AASHTO T283, which is comparable to EN 12697-12 in terms of evaluating the tensile strength ratio of conditioned and unconditioned specimens. Six samples with 7% air void content are used in the test, and these samples are divided into two groups: unconditioned (UC) and conditioned (C). The samples are conditioned at −18 °C for 16 h and then at 60 °C for 24 h, followed by a two-hour holding period at 25 °C, after which the breaking process is performed. The tensile strength (TS) value is calculated using the maximum load value (Eq. 1)^39^:

1 $$\:TS=\frac{2000*P}{\pi\:*t*d}$$

where TS: tensile strength (kPa), P: maximum load value (N), t: height of the specimen (mm), and d: diameter of the specimen (mm).

The tensile strength ratio (TSR) is determined from the ratio of C-TS values to UC TS values. The higher the TSR value, the more resistant the asphalt mixture is to moisture damage^40^.

### Indirect tensile stiffness modulus (ITSM) test

The ITSM test was performed following the test standard EN 12697-26. The stiffness of HMA is related to its capacity to distribute traffic loads and can be considered an indicator of the mixture’s structural properties^41^. The stiffness modulus of the material is a function of the viscoelastic component of the bitumen and is expressed as the ratio between the maximum stress and maximum strain under sinusoidal, uniaxial loading. The modulus of elasticity is a critical performance characteristic used to evaluate the elastic properties of the HMA and measures the deformation of the specimen due to the stress applied to the mixture specimen^42,43^. It also enables the control of the level of stresses that cause deformation of the pavement due to traffic impact. ITSM values of mixture specimens are calculated using Eq. 2^44^.

2 $$\:Sm=\frac{\:L*(\nu\:+\mathrm{0,27})\:}{D*t}$$

where Sm: Indirect tensile stiffness modulus (MPa), L: vertical maximum load applied to the specimen (N), D: average amplitude of horizontal deformation obtained as a result of five load pulses (mm), t: average height of the test specimen (mm), and ν: Poisson’s ratio (normally 0.35 is used).

Accordingly, the experimental parameters were selected as follows: a temperature of 25 °C, a load period of 3000 milliseconds, a load increment time of 124 milliseconds, a target deformation of 5 micrometers, and a Poisson’s ratio of 0.35.

### Indirect tensile fatigue test (ITFT)

The ITFT was performed following the EN 12697-24 standard. Given the inevitable movement of vehicles on asphalt pavements, these surfaces are subjected to short-term loads. As asphalt pavements undergo a loss of hardness, micro-damage accumulates, leading to long-term deformations. The fatigue cracking of asphalt pavements occurs in two stages. The initial stage transpires within the HMA, leading to the formation of a network of micro-cracks that diffuse over time. Subsequently, at a specific micro-crack level (i.e., the fatigue level), the micro-cracks coalesce to form a macro-crack, which then propagates through the material. The theoretical underpinnings of these two stages, designated as crack initiation and propagation, respectively, involve disparate theoretical approaches^45^.

The fatigue life of a specimen is defined as the number of cycles of load applied to the specimen that results in failure or permanent vertical deformation of the specimen. This is determined using the stress values given in Eq. 3 or strain values given in Eq. 4^46^.

3 $$\:Nf=A*{\left(\frac{1}{\sigma\:}\right)}^{n}$$

4 $$\:Nf=\alpha\:*{\left(\frac{1}{\epsilon\:}\right)}^{b}$$

where Nf: number of load cycles representing fatigue life, σ: applied stress, ɛ: initial strain, A, n, ɑ, and b: regression coefficients (fatigue parameters) related to the properties of the mixture.

The crack propagation rate (velocity) (r_p_) is defined as the ratio between the number of load cycles required for each 1 (one) mm deformation to occur after the first crack occurs in the specimen. Equation 5 expressing the crack propagation rate (velocity) (r_p_) is given below^47^.

5 $$\:rp=\frac{\:Np\:}{\delta\:f-\delta\:i}$$

where r_p_: crack propagation rate (speed) (cycles/mm), Np: number of load cycles required for crack propagation, δf: total deformation at the end of fatigue life (mm), δi: total deformation at crack initiation (mm).

In the experiment performed at 25 °C, a loading period of 1.5 s was selected with a load duration of 0.124 s and a rest period of 1.376 s.

### Dynamic creep test

The dynamic creep test is a method of evaluating the resistance of hot mix asphalts to permanent deformation. The resulting deformation varies according to the wave type of the applied loading (impact) as well as the compressive force applied to the specimen. Impact waveforms, including both square and sinusoidal shapes, are frequently employed in dynamic creep testing due to their resemblance to the loads exerted on pavement surfaces by vehicles. The width of the impact loading and the duration of rest directly influence the deformation that occurs. The primary objective of this test is to compare the resistance of various asphalt mixtures to permanent deformation.

The creep modulus value obtained from the test serves as an indicator of resistance to permanent axial deformation, with a low creep modulus indicating a higher propensity for deformation. For specimens with known initial height, at a given temperature and loading time, the creep modulus can be calculated using Eq. 6^48^.

6 $$\:Smix=\frac{\:\:\sigma\:\:\:}{\epsilon\:\:}$$

where Smix: is the creep modulus of the specimen, σ: is the stress applied to the specimen, and ε(:_) is_ the axial strain in the specimen.

The outcomes derived from repetitive creep tests are generally illustrated as the cumulative permanent strain of the specimen in relation to the number of loading cycles. The curve representing cumulative permanent strain is divided into three distinct regions: the first, second, and third. The cycle count at which flow begins in the third region is referred to as the flow number (FN)]. There are several approaches to determining the flow number of HMAs. One such method was developed by Bausano and Williams (2007), and it is as follows: the number of load repetitions that yield the maximum value (peak point) of the curve drawn with the number of load repetitions on the horizontal axis and the product of the creep modulus and the number of load repetitions on the vertical axis is considered the flow number^49^.

## Results and discussions

### Marshall stability and flow test results

Marshall stability and flow tests were conducted on the HMA samples to ascertain the maximum resistance of the mixture specimens to deformation, as well as the extent of deformation that occurred in the specimens upon the application of the maximum load. This analysis was utilized to evaluate the mixtures’ resistance to the formation of permanent deformation. As mentioned in the methodology section, the conditioned specimens were named “C” and the unconditioned specimens were named “UC”.

**Fig. 3:**
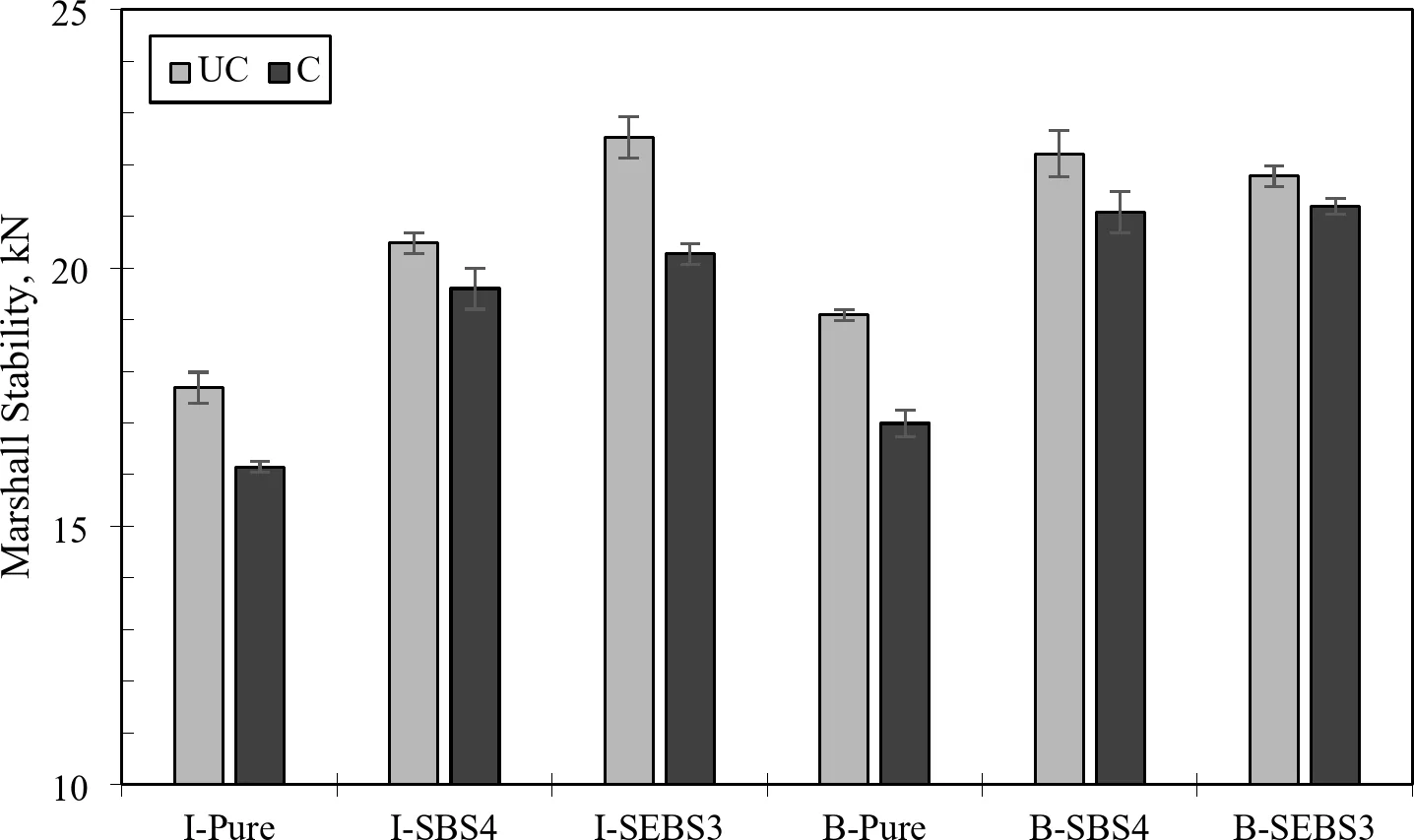
Marshall stability test results.

**Fig. 4:**
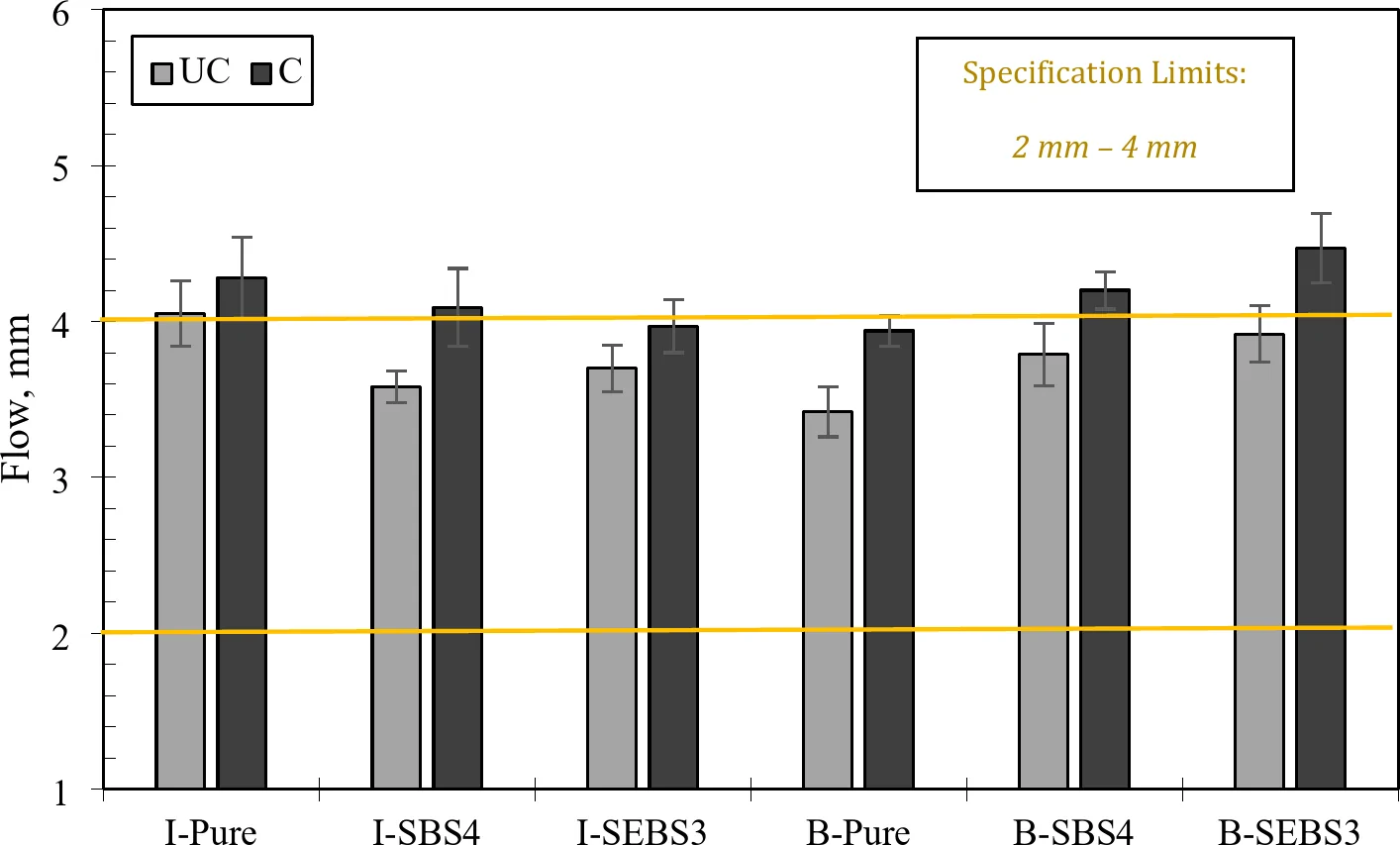
Flow values of mixture specimens obtained from different bitumen.

As illustrated in Fig. 3, the Marshall stability values of the mixture increased with the incorporation of polymers, both in conditioned and unconditioned specimens. The effectiveness of SEBS modification on the stability values was observed to be contingent on the type of bitumen utilized. Specifically, SEBS modification was more effective when Iraqi bitumen was used before conditioning, while SBS modification was more effective when Batman bitumen was employed. It is noteworthy that the stability values of all mixtures underwent a decline following the conditioning process. The effectiveness of polymer use generally increased after conditioning. SEBS modification was found to be more effective when both Iraqi and Batman bitumen were used after conditioning. In general, SBS and SEBS additives showed different effects on stability depending on the bitumen type. SBS modification before conditioning was more effective in increasing stability, especially in Batman bitumen (16.3%), indicating the resistance of SBS to deformation by providing high mechanical strength. Conversely, SEBS modification exhibited a higher increase in stability, 27.4% in the case of Iraqi bitumen, suggesting an enhancement in crack resistance through increased flexibility. Following conditioning, the impact of SEBS became more evident in both bitumen types, surpassing SBS in terms of stability enhancement, with values of 25.5% in Iraqi bitumen and 24.7% in Batman bitumen. This outcome can be attributed to the inherent properties of SEBS, which enable its ability to maintain flexibility under environmental influences, such as conditioning, while concurrently providing a more stable structure against oxidation. A comparative analysis revealed that SBS exhibited higher effectiveness in the Batman bitumen specimens, while SEBS demonstrated superior performance in the Iraqi bitumen specimens. However, the long-term stability maintenance capacity following conditioning was found to be a more significant advantage of SEBS. The flow values of conditioned and unconditioned mixtures for both binder types are depicted in Fig. 4.

**Fig. 5:**
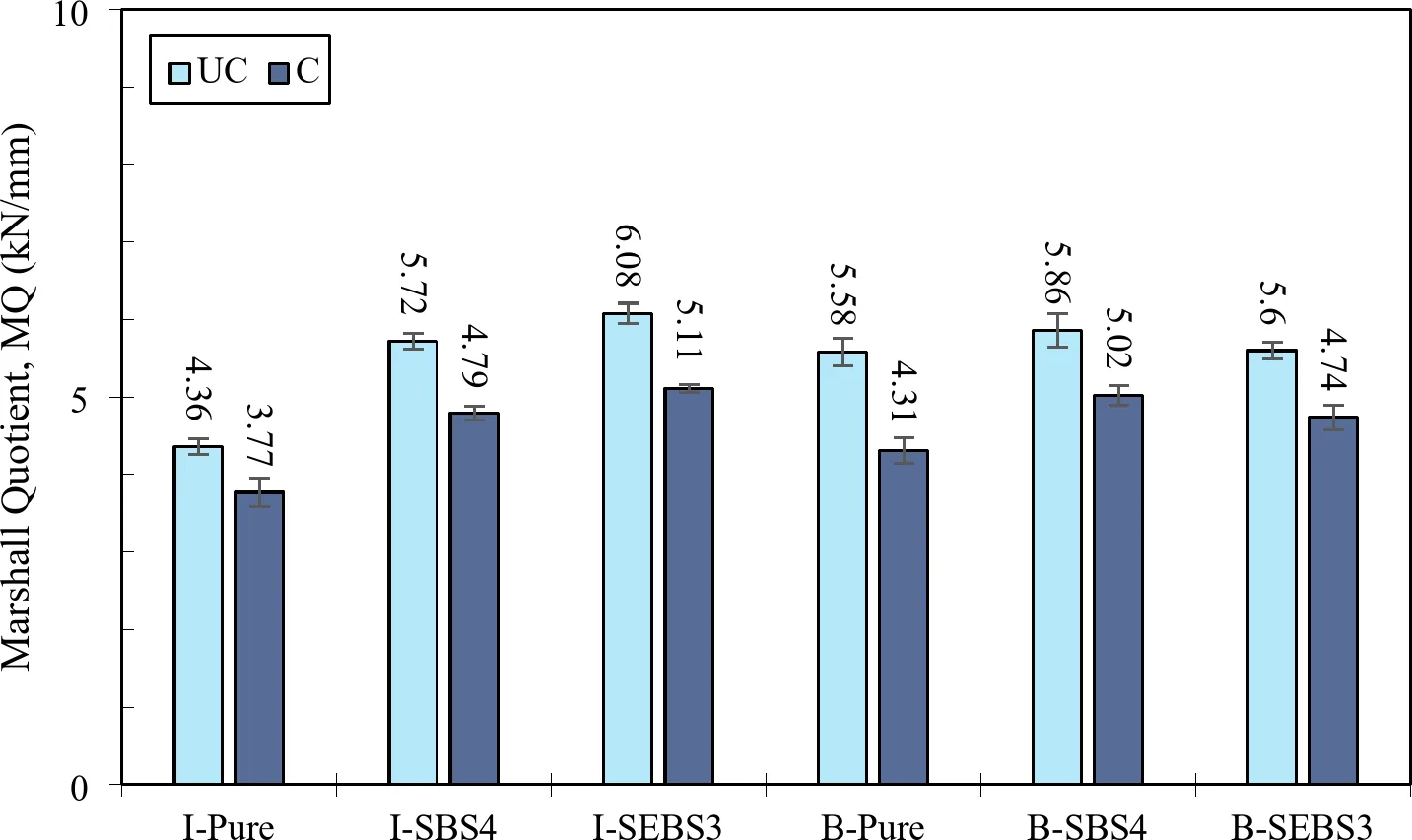
MQ values of the mixtures.

As illustrated in Fig. 4, the flow values of various asphalt mixtures are compared for conditioned (C) and unconditioned (UC) specimens. The conditioned specimens exhibited higher flow values for all mixtures, indicating that conditioning has a negative effect on deformation resistance. While the I-Pure and B-Pure mixtures demonstrated comparable results, the SBS modification (I-SBS4 and B-SBS4) enhanced the deformation resistance, particularly before and after conditioning. SEBS mixtures (I-SEBS3 and B-SEBS3) exhibited a more stable structure with less loss in performance after conditioning, suggesting enhanced durability under environmental influences. According to the standards of the General Directorate of Highways (KGM) in Turkey^50^, I-Pure was the only unconditioned specimen that failed to meet the specified flow value of 2–4 mm. I-SEBS3 and Pure were determined as conditioned specimens meeting the specification limits. In general, it can be said that SBS and SEBS additives increase deformation resistance, but SEBS shows superior performance after conditioning. These findings suggest that the use of mixtures with SBS and SEBS additives will be advantageous, especially under conditions of heavy traffic and high temperatures. The Marshall Quotient (MQ) (kg/mm) is obtained by using Marshall stability and flow values, and the MQ value represents the resistance of the flexible pavement against permanent deformations during its service life (Fig. 5).

The Marshall Quotient (MQ) is a performance parameter that evaluates the stability (load-carrying capacity) and flow (deformation) characteristics of asphalt mixtures. The MQ value is obtained by dividing the stability by the flow, with a higher MQ value indicating that the mixture is more resistant to deformation and deforms less under load. Figure 5 shows the MQ values of conditioned (C) and unconditioned (UC) pure and modified binders. When Fig. 5 is examined, the MQ values of C samples are higher than those of UC samples. No significant difference was observed in the MQ values of mixtures containing pure Iraqi and Batman bitumen. The use of polymer additives resulted in a significant increase in MQ values in both C and UC samples. When comparing polymer additives, SEBS-containing mixtures experienced less strength loss after conditioning compared to SBS-containing mixtures. This was interpreted as indicating that SEBS is more resistant to the conditioning process.

The SBS polymer improved the load-bearing capacity by offering high stability, whereas the SEBS exhibited exceptional performance under various environmental conditions, including conditioning.

**Fig. 6:**
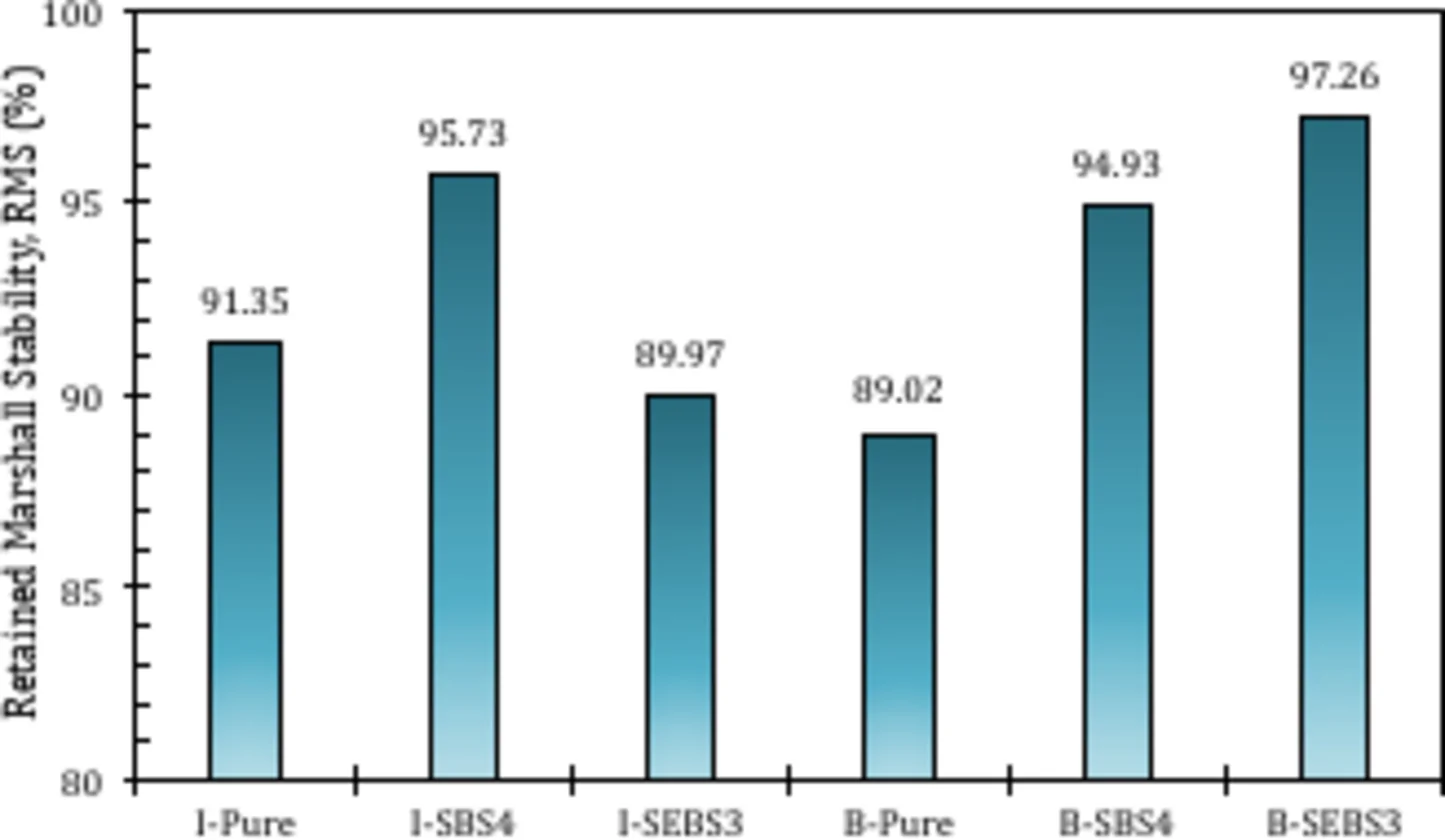
Retained Marshall stability values of the mixtures.

The differences in the retained Marshall stability (RMS) values, which serve as an indicator of the resistance of hot mix asphalts to moisture damage, are derived by comparing the stability values of conditioned mixes to those of unconditioned mixes, and are influenced by binder type and additive usage, as illustrated in Fig. 6. As shown in Fig. 6, the application of Iraqi bitumen led to a significant improvement in the moisture damage resistance of hot mix asphalts, especially when 4% SBS was utilized as a modifier. Conversely, comparable RMS values were achieved with a 3% SEBS modification. For Batman bitumen, it was noted that the RMS values of the mixtures increased markedly with both 4% SBS and 3% SEBS, indicating a general enhancement in the mixtures’ resistance to moisture damage.

An evaluation of the Marshall stability and flow test results revealed that, for the mixtures prepared using Iraqi bitumen, 3% SEBS modification exhibited superior efficacy in mitigating permanent deformation formation before and after conditioning when compared with 4% SBS modification. Conversely, 4% SBS modification demonstrated greater effectiveness in preventing moisture damage formation. For the mixtures prepared using Batman bitumen, 4% SBS modification exhibited superiority over 3% SEBS modification in terms of performance against permanent deformation formation before and after conditioning, while 3% SEBS modification demonstrated superiority over 4% SBS modification in terms of performance against moisture damage formation. Among the prepared mixtures, the mixture prepared using 3% SEBS modified Iraqi bitumen exhibited the most superior performance against permanent deformation formation, while the mixture prepared using 3% SEBS modified Batman bitumen exhibited the most superior performance against moisture damage formation.

### Resistance to moisture damage (tensile strength ratio) test results

**Fig. 7:**
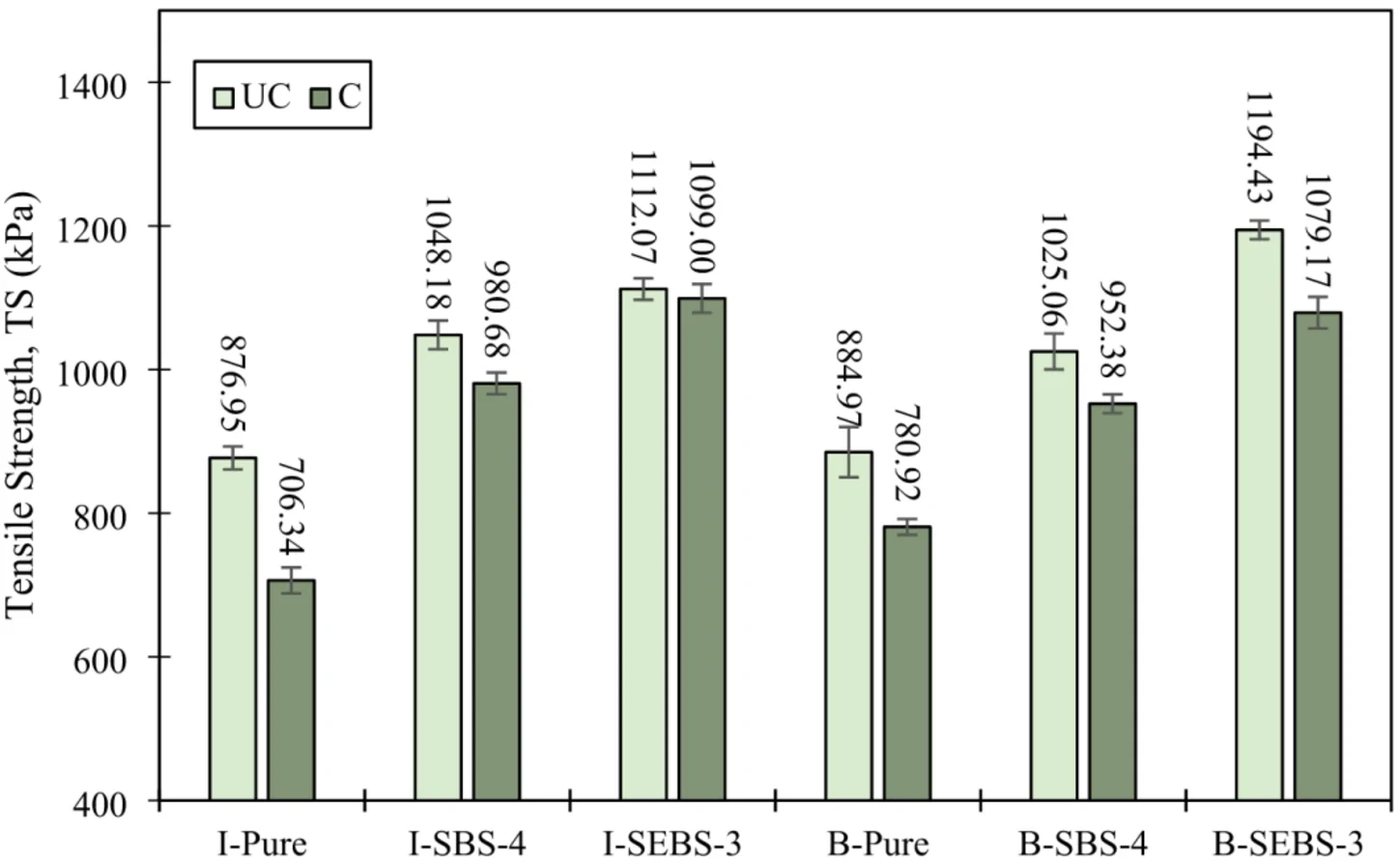
Tensile strength values of mixtures before and after conditioning.

In the present study, the tensile strength ratio (TSR) of unconditioned (UC) and conditioned (C) hot mix asphalt specimens was examined to evaluate their resistance to moisture damage. The TSR values of these specimens are presented in Fig. 7.

**Fig. 8:**
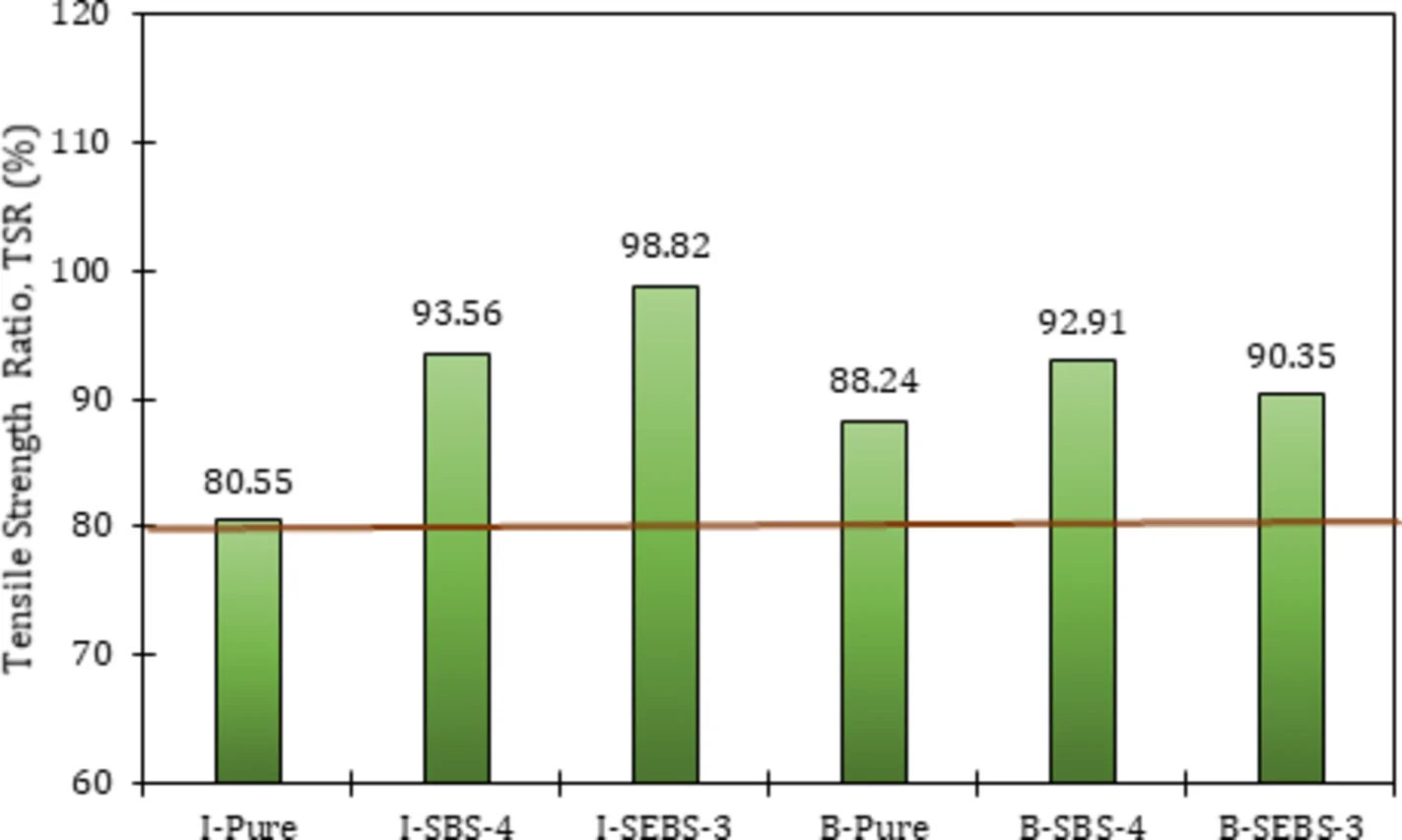
Variations in tensile strength ratios of mixtures.

As demonstrated in Fig. 7, the mixture prepared with pure Iraqi bitumen exhibited the lowest tensile strength value, both before and after conditioning. The conditioning process resulted in a decline in tensile strength values across all mixture types. Significantly, B-Pure exhibited greater tensile strength in comparison to the I-Pure, both prior to and following conditioning. Specifically, the tensile strength of the blend utilizing Batman bitumen was 0.9% superior to that of the Iraqi bitumen blend before conditioning, and this disparity expanded to 10.6% after conditioning. Significant increases in tensile strength were observed in the mixtures using modified bitumen compared to the mixtures prepared with pure bitumen. The tensile strength of the I-SBS4 mixture was found to be 19.5% higher than that of the I-Pure mixture, while the tensile strength of the I-SEBS3 mixture was found to be 12.8% higher. For Batman bitumen, these values are 15.8% and 35%, respectively. Figure 8 shows the tensile strength ratio (TSR) results.

According to Fig. 8, B-Pure exhibited higher resistance to moisture damage compared to I-Pure. However, when modified bitumens were used, the TSR values of the mixtures prepared with Iraqi bitumen were higher than those prepared with Batman bitumen. According to the Superpave method, TSR values of hot mix asphalts should exceed 80%. In this study, it was ascertained that the TSR values of all mixtures prepared were above 80%, thereby demonstrating adequate performance against moisture damage. The highest performance was observed in mixtures prepared with 3% SEBS-modified Iraqi bitumen. In terms of tensile strength (TS) values, it was observed that 3% SEBS modification was more effective than 4% SBS modification. However, in terms of TSR values, 3% SEBS modification was more effective in mixtures prepared with Iraqi bitumen, while 4% SBS modification provided a higher performance in mixtures prepared with Batman bitumen.

### Indirect tensile stiffness modulus (ITSM) test results

**Fig. 9:**
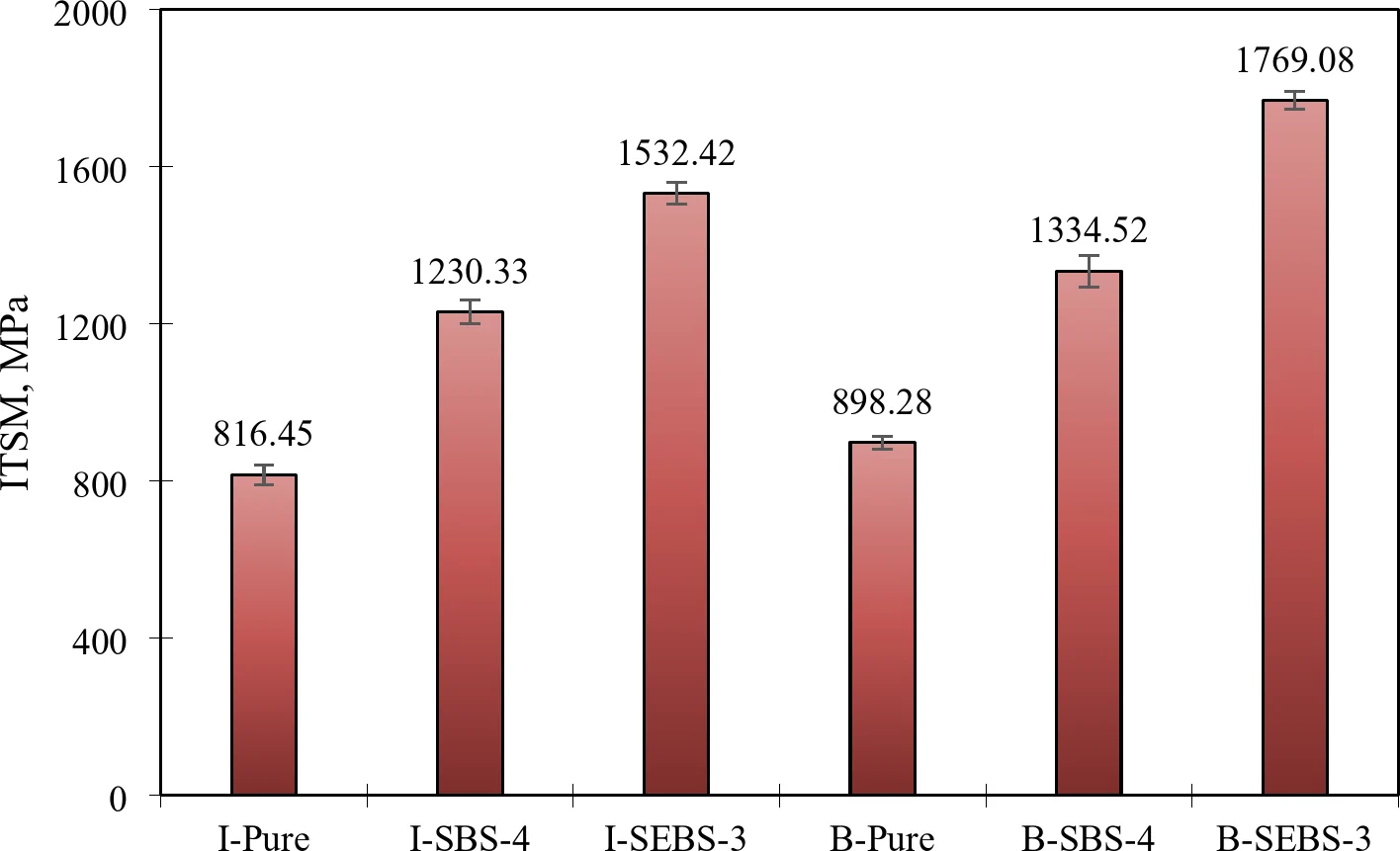
ITSM test results for different bitumen types.

The variations in ITSM values of the mixtures prepared using pure and modified bitumen are illustrated in Fig. 9.

When the ITSM values given in Fig. 9 are examined, it is seen that the B-Pure performs 10% better than I-Pure. The I-SBS4 mixture offers 50.7% higher rigidity values compared to the pure mixture, while this increase is 87.7% in the I-SEBS3 mixture. The B-SBS4 sample shows a 48.6% increase compared to the pure mixture, while the B-SEBS3 mixture shows a 96.9% increase. According to the ITSM results, Iraqi bitumen is more efficient for SBS modification, while Batman bitumen is more efficient for SEBS modification. Other test results also confirm this.

### Indirect tensile fatigue test (ITFT) results

The fatigue life (Nf) of the specimens was ascertained from the intersection of the tangents drawn for regions II and III of the curve, as illustrated in Fig. 1. The tangent for region III of the curve was constructed at the point where the slope exhibits a significant increase, as shown in Fig. 2. The equations for the tangents were formulated, and the fatigue life (Nf) of the specimens was calculated by equating these two tangent equations, as demonstrated in Fig. 3. The boundary point that distinguishes regions II and III of the curve was recognized as the number of load repetitions (Ni) required for the onset of cracks in the mixture specimen. The values for crack propagation (Np) were obtained by subtracting the number of load cycles (Ni) necessary for crack propagation from the fatigue life (Nf) values.

The crack propagation rate (r_p_) values of the mixture specimens were determined by using the crack propagation load number of cycles (Np), total deformation at the end of fatigue life (δf), and total deformation at the point where the crack starts to form (δi).

**Fig. 10:**
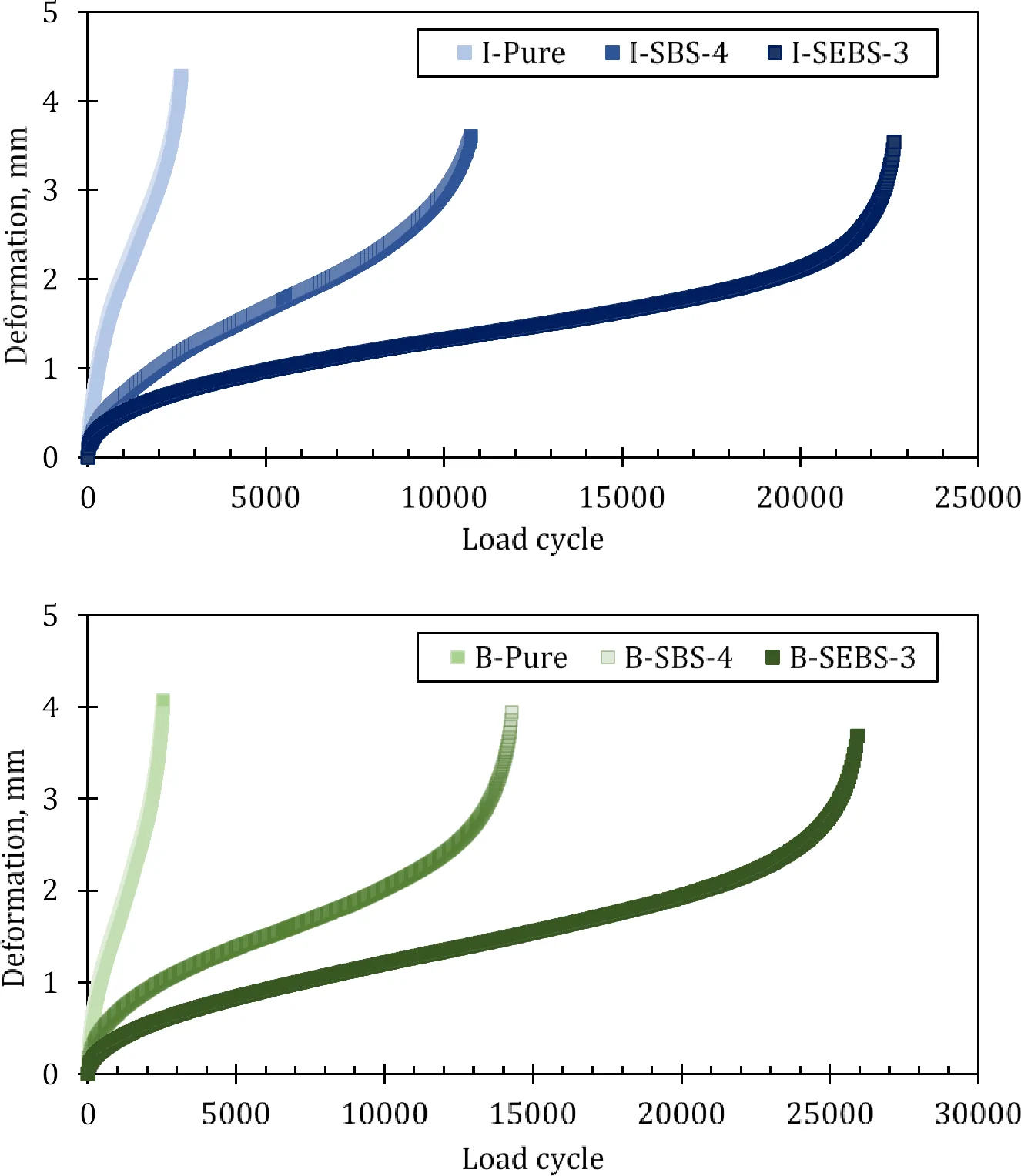
Deformation-load repetition number relationship of mixtures prepared with Iraqi and Batman bitumen.

Figure 10 shows the deformation-load repetition number graphs of pure and modified mixtures prepared using Iraqi and Batman bitumen.

**Fig. 11:**
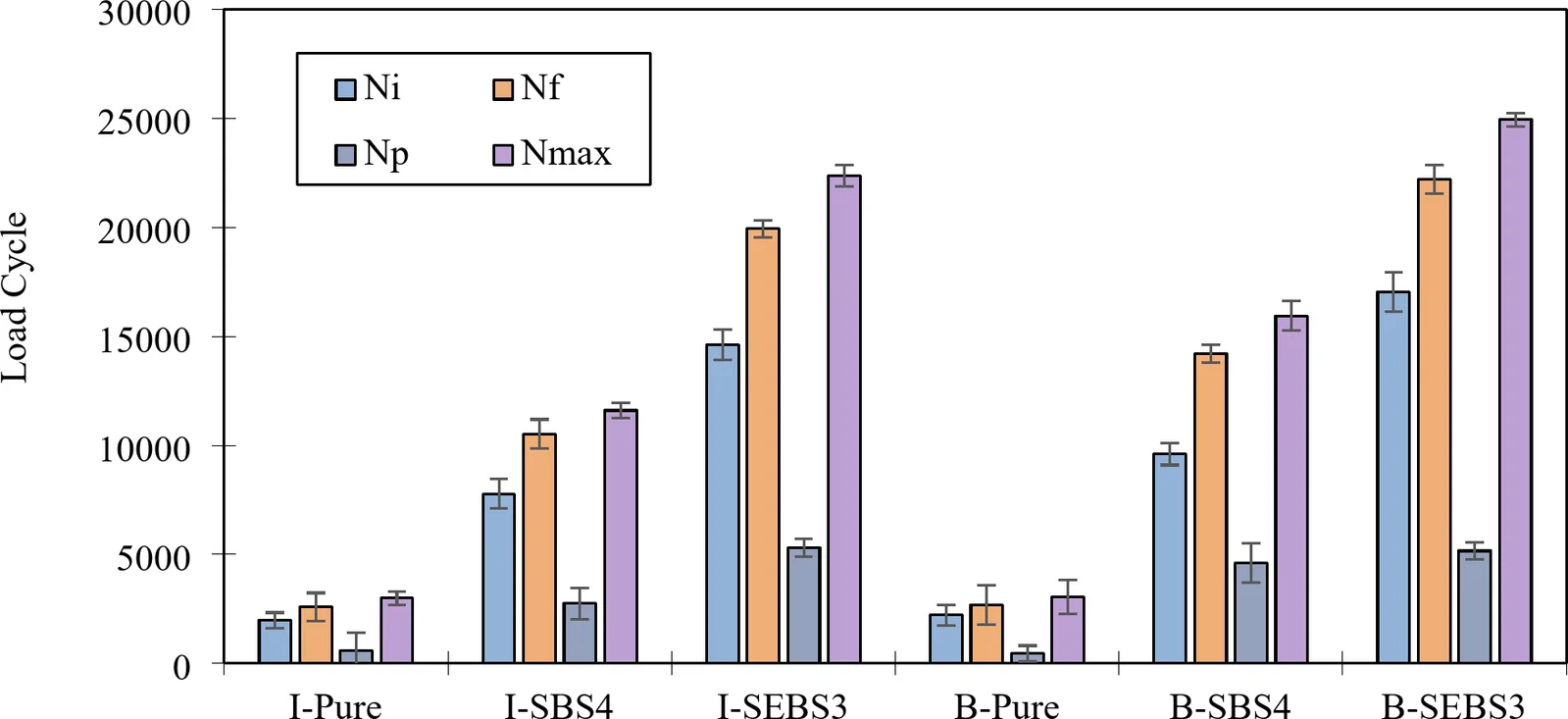
Variations in load repetition number values with binder and additive type.

As demonstrated in Fig. 10, the minimum number of load repetitions necessary for fracture to occur in both the mixture specimens prepared using Iraqi bitumen and the mixture specimens prepared using Batman bitumen was identified. The mixtures created using pure bitumen demonstrated the least number of load repetitions, whereas those made with 3% SEBS-modified bitumen showed the greatest. Further examination of the deformation values indicated a reduction with the addition of additives for both Iraqi and Batman bitumen. Importantly, pure mixtures displayed the highest deformation values, in contrast to the mixtures made with 3% SEBS-modified bitumen, which revealed the lowest. The data acquired regarding the number of load repetitions necessary for crack initiation (Ni), fatigue lifespan (Nf), the number of load repetitions needed for crack growth (Np), and the peak load repetitions (Nmax) are illustrated in Fig. 11. These values were obtained by applying a 250 kPa stress level and using three specimens for each mixture type with the use of SBS and SEBS additives for both Iraqi and Batman bitumen.

As demonstrated in Fig. 11, the Ni, Nf, Np, and Nmax values exhibited an increase with SBS and SEBS modification for both Iraqi and Batman bitumen. It was determined that the Ni value of B-Pure was 11.9% greater, the Nf value was 3.8% greater, and the Nmax value was 2.2% greater compared to I-Pure. Additionally, the crack propagation load repetition number (Np) values for the B-Pure were observed to be 22.9% lower than those for the I-Pure. This finding suggests that while the fatigue life of the mixtures is higher when pure Batman bitumen is used in the production of hot mix asphalts than when pure Iraqi bitumen is used, the mixtures prepared using pure Batman bitumen will reach the end of the fatigue life earlier after the crack formation.

Moreover, it was found that the Ni value of the B-SBS4 was 23.4% greater, the Nf value was 34.9% greater, the Np value was 67.4% greater, and the Nmax value was 37.4% greater in comparison to I-SBS4. A comparable pattern was noted for B-SEBS3, where the Ni value increased by 16.6%, the Nf value rose by 11.4%, the Np value increased by 11.4%, and the Nmax value rose by 11.5% relative to the I-SEBS3. However, the Np value of the B-SEBS3 was − 2.7% lower than I-SEBS3. From the results obtained, it was determined that the use of pure Batman bitumen was more effective than pure Iraqi bitumen in terms of fatigue life, and this effect increased with the modification process. The findings indicate that the fatigue life of hot mix asphalts exhibited a substantial increase with the incorporation of additives, with 3% SEBS modification proving to be the most efficacious additive and ratio. Furthermore, modification with Batman bitumen emerged as a more effective approach compared to Iraqi bitumen.

**Fig. 12:**
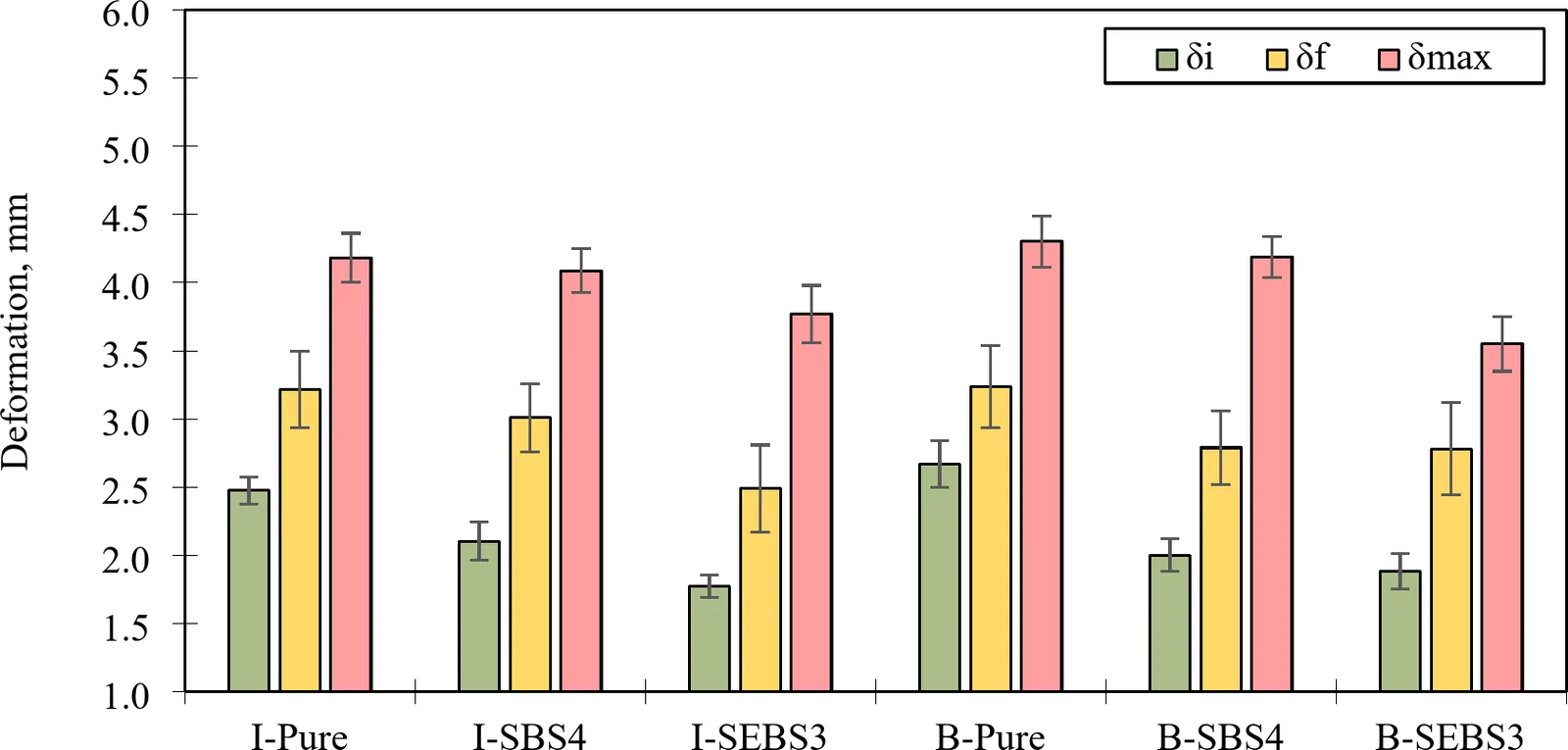
Variations in deformation values with binder and additive type.

The differences in the average total deformation values (δi) at the crack initiation point, the average deformation values (δf) at the fatigue life (Nf), and the average deformation values (δmax) at the peak number of load repetitions of hot mix asphalt specimens utilizing SBS and SEBS additives for both Iraqi and Batman bitumen are illustrated in Fig. 12.

As demonstrated in Fig. 12, the incorporation of additives in the mixtures prepared using both Batman and Iraqi bitumen led to a substantial reduction in deformation amounts when compared to the pure mixtures. Specifically, in the case of Iraqi bitumen, the initial deformation (δi) was reduced by 15.06%, the final deformation (δf) by 6.49%, and the maximum deformation (δmax) by 2.30% for the mixtures prepared with 4% SBS modification. The incorporation of 3% SEBS modification with the same binder resulted in a 28.34% decrease in δi, a 22.62% decrease in δf, and a 9.88% decrease in δmax. Similarly, when Batman bitumen was utilized, mixtures prepared with 4% SBS modification exhibited a 24.98% decrease in δi, a 13.84% decrease in δf, and a 2.58% decrease in δmax. The findings indicate that the deformation values of the mixtures prepared with Batman bitumen are higher than those prepared with Iraqi bitumen. Furthermore, the 3% SEBS modification was identified as the additive that most effectively reduced the deformation amounts. However, it was also noted that there was no regular variation between the mixtures prepared with modified binders. In general, it was determined that the additives for both types of binders reduced the deformation.

**Fig. 13:**
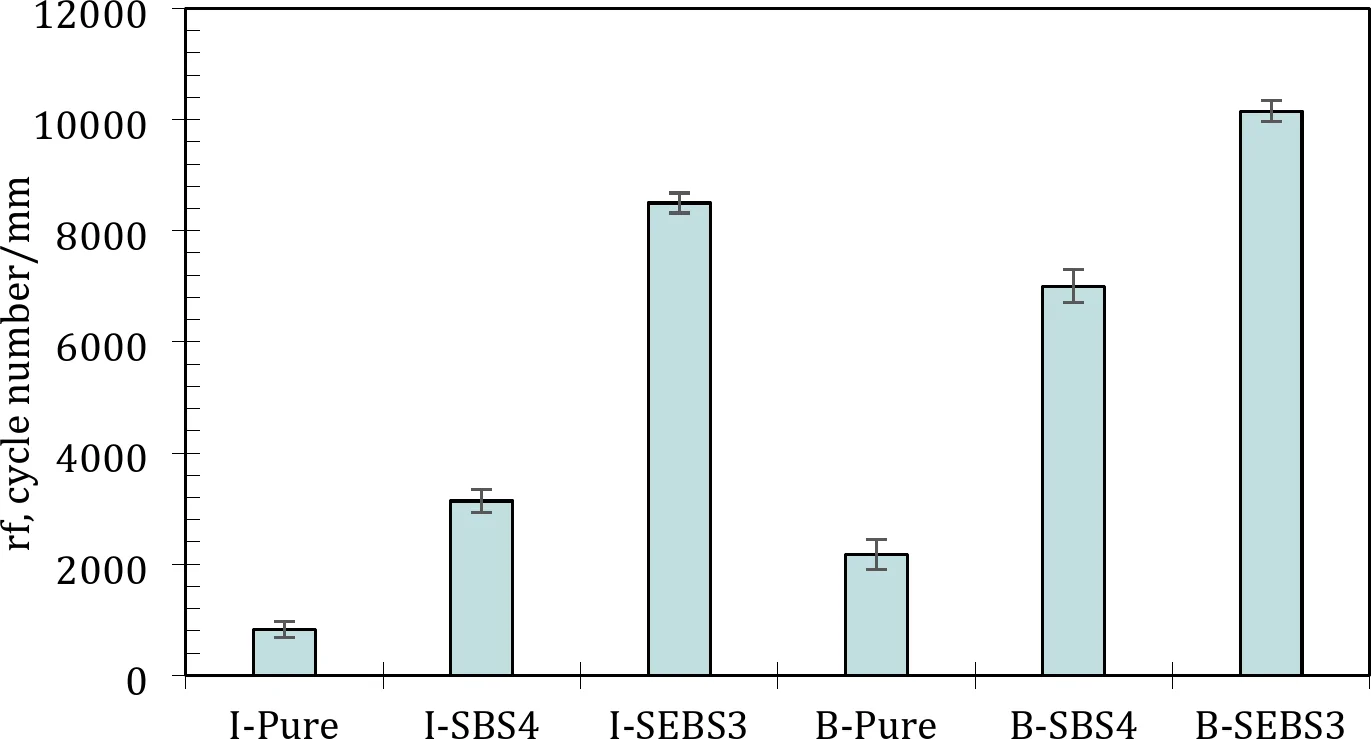
Variations in crack propagation ratio (r_p_) between Ni and Nf with binder and additive type.

The variations in crack propagation rate values (r_p_), which are used to express the number of repetitions of load required for each 1 mm deformation of the specimen after crack formation (N_i_ value) until the fatigue life of the specimen (N_f_ value), with the use of additives for both Iraqi and Batman bitumen, are given in Fig. 13.

An analysis of the rp values obtained from the fatigue test of the mixtures revealed that those mixtures containing pure Batman bitumen exhibited 163.4% higher r_p_ values in comparison to the mixtures containing pure Iraqi bitumen. Similarly, the mixtures containing 4% SBS-modified Batman bitumen demonstrated The mixtures using 4% SBS-modified Batman bitumen exhibited 123.2% higher r_p_ values compared to the mixtures using 4% SBS-modified Iraqi bitumen. Additionally, the mixtures using 3% SEBS-modified Batman bitumen demonstrated 19.4% higher r_p_ values compared to the mixtures using 3% SEBS-modified Iraqi bitumen. Furthermore, it was determined that 3% SEBS modification exhibited superior efficacy in comparison to 4% SBS modification in terms of additive type. Additionally, it was observed that Batman bitumen demonstrated greater effectiveness in terms of binder on the r_p_ values of the mixtures when compared to Iraq bitumen.

### Dynamic creep test results

**Fig. 14:**
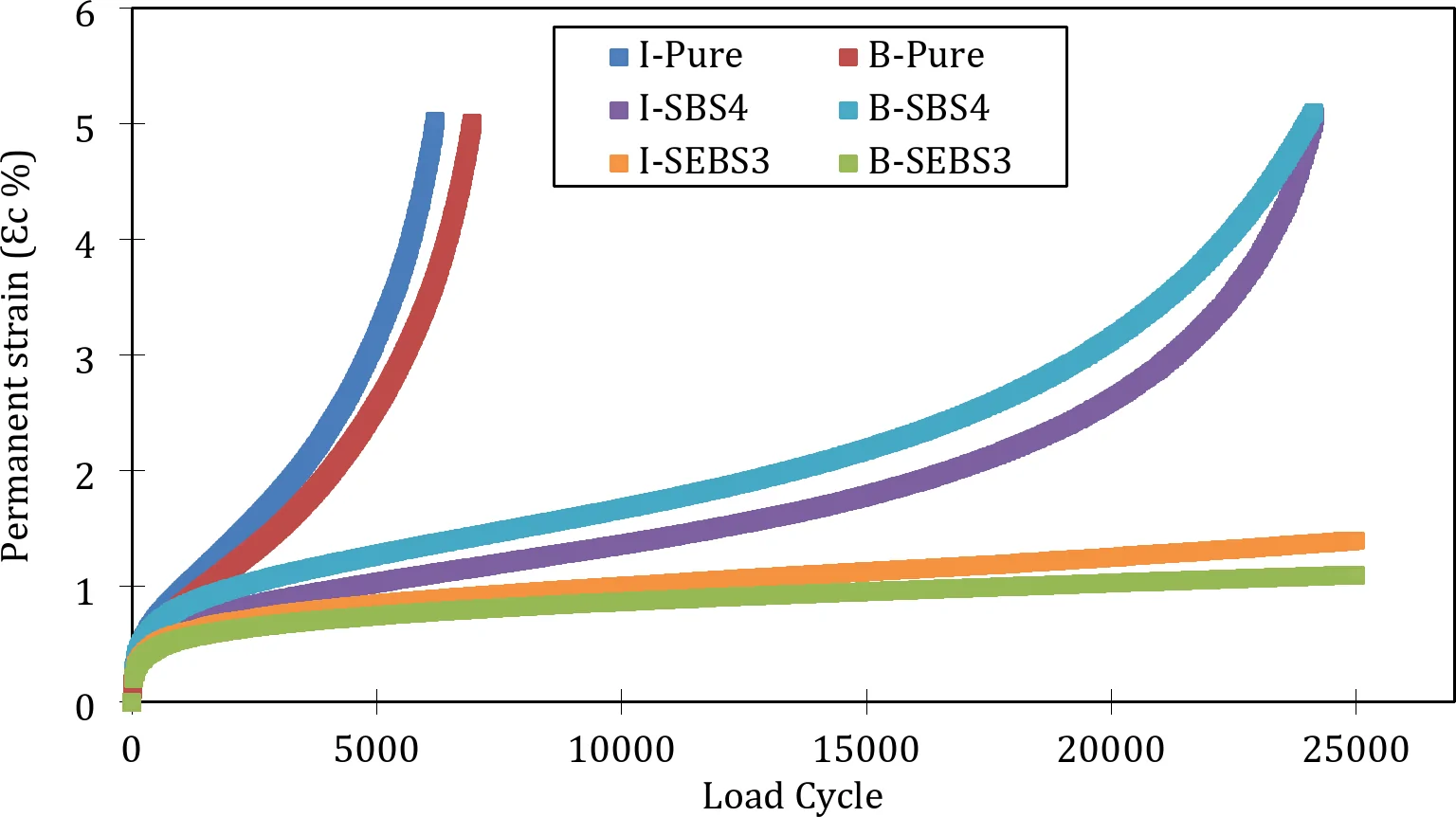
Ɛc - load repetition number relationship of different mixture specimens.

The permanent strain amounts (Ɛ_c_) on different mixture specimens, depending on the number of applied load repetitions, are shown in Fig. 14.

As illustrated in Fig. 14, the number of load repetitions required to induce 5% permanent strain in the mixture specimens prepared using 3% SEBS and 4% SBS is considerably higher than pure samples. In the mixture specimens prepared using 3% SEBS, it was determined that even at 25,000 load repetitions, the 3rd region, which is accepted as the region where the flow starts on the specimen, was not passed and a small amount of deformation occurred compared to pure and SBS modified samples.

**Fig. 15:**
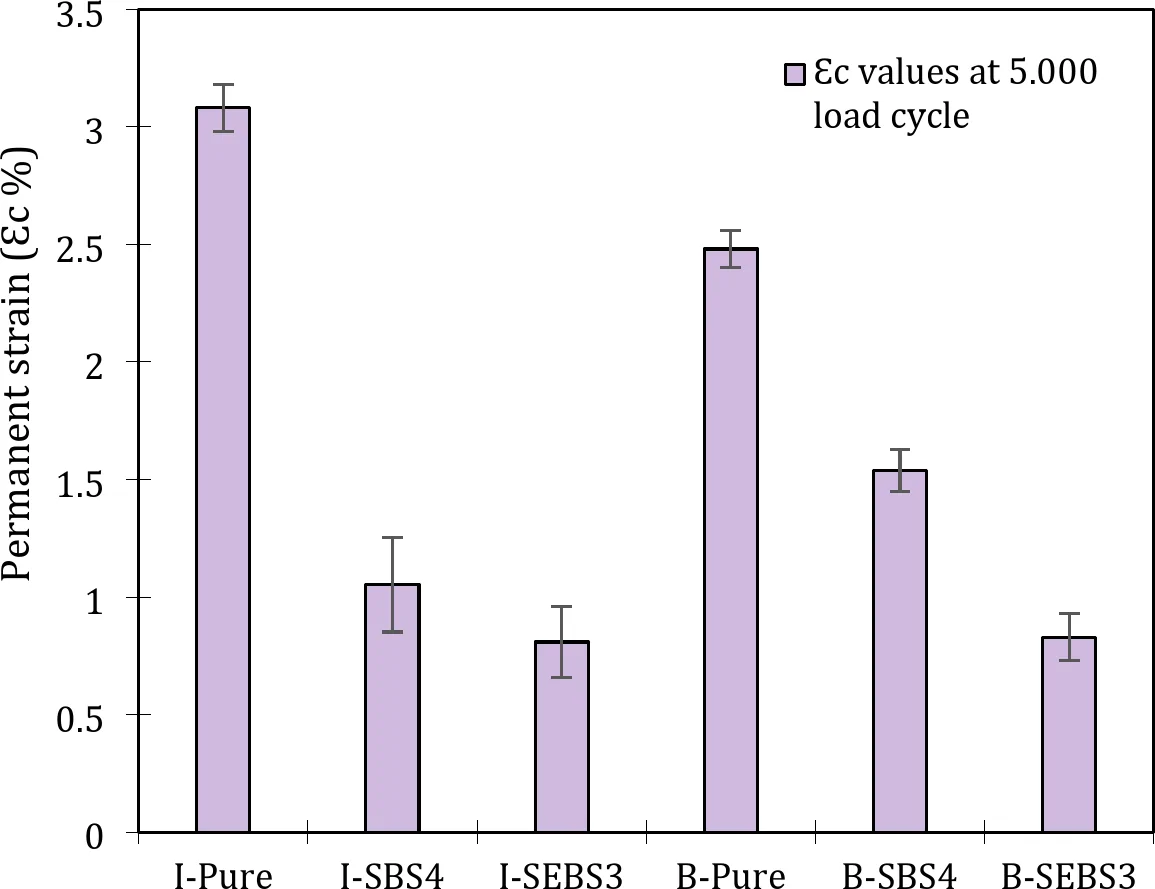
Ɛc values of the mixtures after 5,000 load repetitions.

Figure 15 presents the permanent strain values of all mixtures following 5,000 load repetitions, as applied to the mixture specimens. These values were derived using the mean of three specimens prepared for each mixture type within the scope of the creep test.

As seen in Fig. 15, the incorporation of SBS and SEBS additives into pure Iraqi and pure Batman bitumen resulted in a substantial reduction in permanent strain values. At a load repetition of 5,000, the Ɛ_c_ value of the B-Pure was found to be lower than the I-Pure. This outcome suggests that B-Pure exhibits higher resistance to permanent deformation compared to the I-Pure. For both mixture types, it was determined that SEBS modification was more effective than SBS modification, resulting in lower permanent strain values on the mixture specimens. Upon examining the permanent strain values of the mixture specimens created with Iraqi bitumen after 5,000 load repetitions, it was found that the Ɛc value of the mixture formulated with 4% SBS modified Iraqi bitumen diminished by a factor of 2.93 in comparison to the Ɛc value of the mixture made with pure Iraqi bitumen. Additionally, the Ɛc value of the I-SEBS3 showed a reduction of 3.81 times relative to the I-Pure. Furthermore, when analyzing the Ɛc values of the mixture specimens prepared with Batman bitumen after 5,000 load repetitions, it was observed that the Ɛc value of the B-SBS4 decreased by 1.61 times compared to the B-Pure, while the Ɛc value of the B-SEBS3 decreased by 2.99 times in relation to the B-Pure. Upon evaluation of these results, it is determined that Iraqi bitumen is more effective than Batman bitumen in both SBS and SEBS modification on permanent strain values. However, B-Pure has lower values than I-Pure in terms of permanent strain value.

**Fig. 16:**
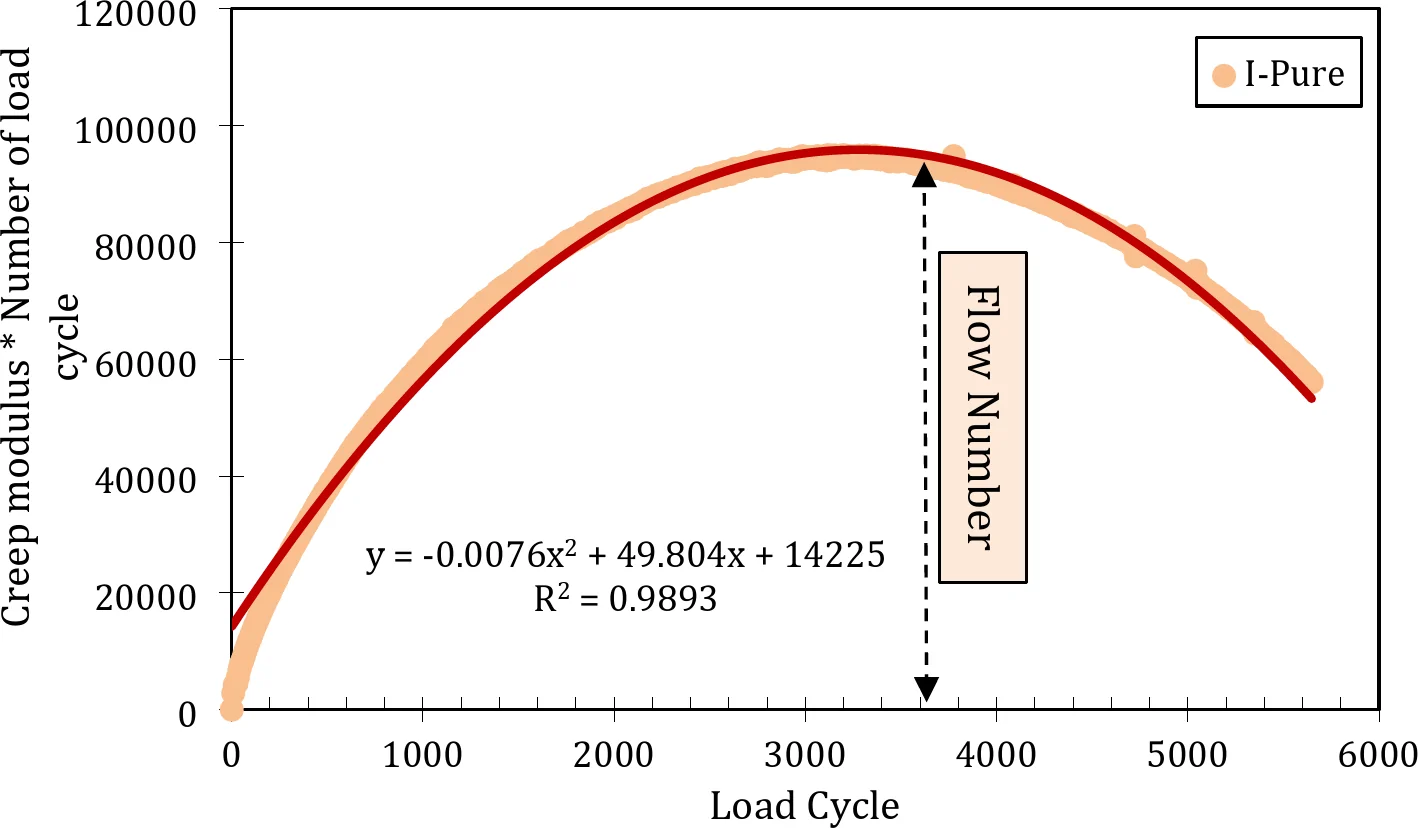
Illustration of the flow number calculation.

**Fig. 17:**
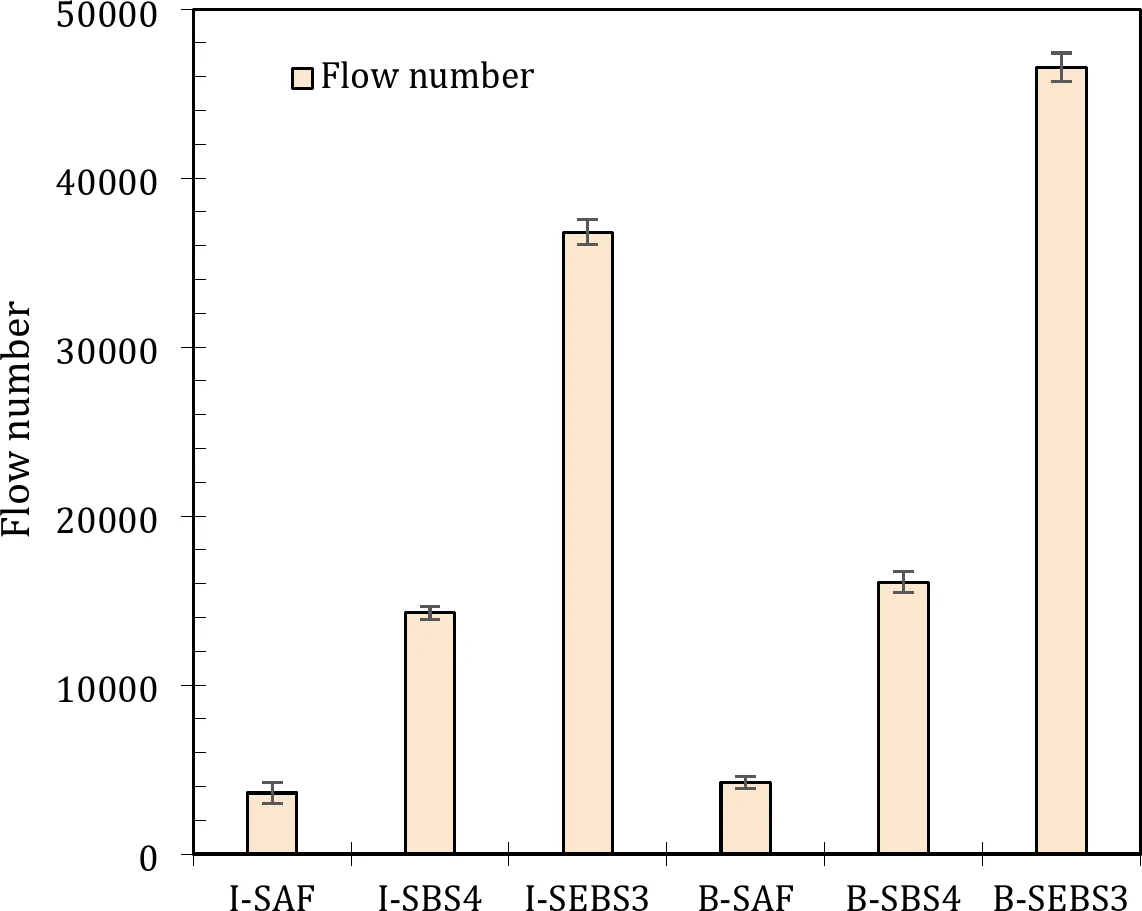
Flow numbers of the prepared mixtures.

To ascertain the flow number values of the specimens utilized in the present study, the methodology proposed by Bausano and Williams (2007) was employed. This methodology involves the expression of the flow number as the number of load repetitions that correspond to the maximum point of the curve, which is drawn against the load repetition number values on the horizontal axis and the product of the load repetition number and creep modulus values on the vertical axis^49^. As an illustrative example, Fig. 16 presents the curve drawn against the number of load repetitions of the I-Pure mixture specimens, the curve drawn against the product of the number of load repetitions and the creep modulus values, and the second-degree polynomial trend line of this curve and the equation of the trend line. The maximum point of the trend line corresponds to the value that makes the derivative of the trend line equation zero. This value is the number of load repetitions expressing the flow number. Figure 17 shows the flow numbers of six different mixture types prepared within the scope of the study.

When Fig. 17 is examined, it can be seen that the mixture containing Batman bitumen is 16.8% higher than that of Iraq. B-SBS4 samples provided 12.7% higher flow number values than I-SBS4. B-SEBS3 samples provided 26.5% higher flow number values than I-SEBS3. Overall, when evaluated in terms of flow number, it was determined that Batman bitumen performs better for both pure and modified binders.

Binders with similar penetration grades may still differ significantly in their chemical composition and colloidal structure depending on the crude oil source. Variations in aromatic content, asphaltene distribution, and resin fractions influence polymer compatibility, swelling behavior, and phase structure, which in turn affect the mechanical performance of polymer-modified mixtures. Therefore, the observed differences between the Batman and Iraqi binders can be attributed to source-dependent binder characteristics, as also reported in previous studies.

Polymer modification has been widely used to enhance the performance of asphalt mixtures, with SBS being the most commonly applied modifier due to its ability to form a three-dimensional elastic network that improves stiffness, elasticity, and resistance to deformation. However, SBS is known to be susceptible to oxidative and thermal degradation due to the presence of unsaturated double bonds in its structure. In contrast, SEBS, which is obtained by hydrogenation of SBS, possesses a saturated molecular structure that provides superior resistance to aging, oxidation, and environmental effects. As a result, SEBS-modified mixtures generally exhibit improved durability, moisture resistance, and long-term performance. Previous studies have shown that SEBS modification can significantly enhance water damage resistance, tensile properties, and fatigue performance of asphalt mixtures, primarily by improving binder–aggregate adhesion and promoting a more stable polymer network structure^51^. Moreover, SEBS has been reported to increase fracture energy and energy absorption capacity compared to SBS under certain loading conditions, indicating enhanced resistance to crack initiation and propagation^1^. In addition, SEBS-modified mixtures have demonstrated superior moisture susceptibility performance and mechanical stability compared to conventional SBS-modified mixtures, particularly at optimal polymer contents^52^. These findings support the results of the present study, where SEBS modification led to improved mixture performance, although the extent of improvement was found to depend on the base binder characteristics.

## Conclusions

In this study, the SBS and SEBS modification efficiencies of bitumen obtained from two different sources with the same penetration degree were investigated in a systematic laboratory study. The results obtained in the study are summarized below.

- According to the results of the Marshall Stability and Flow test, polymer modification significantly improved mechanical performance. The I-SEBS3 mixture performed better in terms of stability, while the B-SEBS3 mixture performed better in terms of flexibility. For Iraqi bitumen, a 3% SEBS addition, and for Batman bitumen, a 4% SBS addition were the most effective mixtures.
- According to moisture damage resistance results, the I-SEBS3 mixture provided the highest TSR value, while a 4% SBS addition for Batman bitumen yielded the highest TSR value.
- ITSM results showed that both polymer additives consistently increased stiffness values. The highest ITSM value was obtained in the B-SEBS3 mixture. Under indirect tensile load, SEBS provided higher performance than SBS for both binders.
- The fatigue behavior of the mixtures was investigated using ITFT, and it was determined that the polymer additives significantly increased the fatigue and cracking performance of the mixture samples. A 3% SEBS addition provided higher fatigue performance compared to a 4% SBS addition, with the highest fatigue performance obtained in the B-SEBS3 sample.
- The dynamic creep test demonstrated that the polymer additive provides resistance to permanent deformation. For both binders, the 3% SEBS modification provided better results compared to 4% SBS, with the highest flow number value obtained in the B-SEBS3 mixture.

This study focuses on performance-based evaluation; therefore, detailed chemical analyses such as SARA fractionation were not conducted. Polymer modification (SBS, SEBS) is primarily associated with physical interactions rather than significant changes in chemical fractions. Nevertheless, SARA analysis could provide additional insight and is recommended for future studies. DSR testing in this study was limited to temperature sweep for PG determination. Frequency sweep and master curve analyses were not included, as the focus was on mixture performance; however, such analyses may be considered in future work.

## Data Availability

The datasets generated during and/or analysed during the current study are available from the corresponding author on reasonable request.
